# QPRT Acts as an Independent Prognostic Factor in Invasive Breast Cancer

**DOI:** 10.1155/2022/6548644

**Published:** 2022-02-24

**Authors:** Lili Zhou, Lin Mu, Wenyan Jiang, Qi Yang

**Affiliations:** Department of Radiology, First Hospital of Jilin University, Changchun, Jilin 130031, China

## Abstract

**Background:**

Quinolinic acid phosphoribosyltransferase (QPRT) is a rate-limiting enzyme that encodes the uronic acid pathway, which is involved in cell cycle progression and cancer cell metabolism. Some studies have demonstrated the progrowth effect of QPRT on breast cancer (BRCA) tumour cells, but its mechanism of action requires further exploration.

**Methods:**

We investigated the expression of QPRT and the prognosis of patients with different tumours by performing a pan-cancer analysis of QPRT. Prognostic values for overall survival (OS) were determined using uni- and multivariate Cox proportional hazard analyses. The prognostic survival of patients with a different pathological staging of BRCA and with QPRT high and low expression was also analysed. We also explored the relevant pathways by which QPRT affected BRCA tumorigenesis by gene set enrichment analysis (GSEA) and western blotting. The impact of QPRT on the PI3K/Akt pathway was also evaluated.

**Results:**

Pan-cancer analysis revealed significant QPRT expression in pan-cancer and correlated with prognosis in most tumour patients. QPRT was also highly expressed in BRCA when patients had poor prognoses, and its expression was associated with different pathological BRCA subtypes. GSEA revealed an association between BRCA progression and the cell cycle and the phosphatidylinositol 3-kinase (PI3K)/Akt signalling pathway, and this association was confirmed by western blotting.

**Conclusion:**

QPRT is highly expressed in breast cancer and particularly in HER2 breast cancer. Upregulated QPRT expression is an independent predictor of breast cancer prognosis and promotes breast cancer progression by activating the PI3K/Akt signalling pathway.

## 1. Introduction

As of 2020, invasive breast cancer (BRCA) (2.26 million cases) remains one of the most commonly diagnosed cancer types worldwide [1] and is the leading cause of cancer death in women aged 20–59 years [2]. Currently, standard screening methods for BRCA include mammography, magnetic resonance imaging (MRI), computed tomography (CT), and biopsy [3]. Despite significant advances in diagnostic tools and treatment strategies, BRCA continues to rise in prevalence and affects approximately one in twenty countries worldwide [4], with higher rates in developed countries [5]. BRCA is classified, based on differences in gene expression patterns, into five major categories, luminal A, luminal B, HER2 overexpression, basal_like, and normal_like, with HER2 overexpressing breast tumours having the poorest prognosis [6]. BRCA is metastatic cancer and can spread to distant organs, such as the bone, liver, and lung, a condition that is often incurable, whereas early diagnosed BRCA generally has a better prognosis and survival rate [7]. The 5-year survival rate of patients with stage I BRCA can be as high as 100%, while in patients with stage IV BRCA, it decreases to 26% [8].

In BRCA cells, a reduction in cellular levels of nicotinamide adenine dinucleotide (NAD+) may induce apoptosis [9]. NAD+ is a critical coenzyme involved in the redox reactions of cancer cell metabolic pathways [10] and plays a role in DNA repair, gene transcription regulation, the cell cycle, apoptosis, metabolism, and other biological processes [11]. The production of NAD+ is promoted by the activity of quinolinate phosphoribosyltransferase (QPRT), the rate-limiting enzyme encoding the kynurenic pathway, via catabolism tryptophan. QPRT is upregulated in cancer cells, and this upregulation is resistant to inhibitors of nicotinamide phosphoribosyltransferase (NAMPT) [12], the rate-limiting enzyme of the NAD + salvage pathway [13]. Studies have shown that high expression of NAMPT is related to the aggressive biological characteristics of BRCA [14] and can regulate the PI3K-AKT signalling pathway and promote tumour cell proliferation [15]. Similarly, QPRT expression has shown great relevance to the migration and invasive ability of BRCA cancer cells [16], and tumour xenograft assays have demonstrated the growth-promoting effect of QPRT overexpression in BRCA tumour cells [17].

The mechanism of action of QPRT on BRCA onset and progression has not been adequately studied. Therefore, the present study aimed to analyse the impact of QPRT on the expression and prognosis of BRCA tumours. The biological functions and pathways of QPRT were studied through gene set enrichment analysis (GSEA), the relationship between QPRT and signalling pathways was established by western blot analysis, and the mechanism of QPRT effects on breast cancer progression was determined.

## 2. Materials and Methods

### 2.1. Data Sources and Processing

Data were obtained from The Cancer Genome Atlas (TCGA) dataset, Cancer Cell Line Encyclopedia (CCLE), and Genotype-Tissue Expression (GTEx) dataset, including clinical information data and gene expression matrices for normal tissues, tumour tissues, and tumour cell lines. In total, 33 tumour samples were obtained from the TCGA dataset, RNA sequencing data for 21 tumour cell lines were obtained from the CCLE dataset, and expression profile data were obtained for 27 cancer and paracancer tissues by integrating the TCGA and GTEx datasets. The expression matrices of GSE46563 and GSE59246 were obtained from the GEO database (https://www.ncbi.nlm.nih.gov/geo/). GSE46563 contains 75 HER− and 19 HER+ samples, and GSE59246 contains a total of 50 HER− and 19 HER+ samples.

### 2.2. Analysis of QPRT Expression and Survival in Each Tumour

The Kruskal–Wallis test was used to analyse differences in tumour tissues and normal tissues. Survival analysis was performed using the R survival package, and the disease-specific survival (DFS) between QPRT expression and patients with different tumours was examined using one-way Cox risk proportional regression analysis, with data expressed as forest plots. Kaplan–Meier (KM) analysis was used to test the association between QPRT and survival among patients with different tumours. A value of *P* < 0.05 was considered statistically significant.

### 2.3. Expression and Survival Analysis of QPRT in BRCA

The differences in QPRT expressions in different pathological subtypes of BRCA were analysed using the R package Limma. The log-rank test was used to test the survival differences between the high and low QPRT expression groups, and KM curves were plotted to show the overall survival (OS) and progression-free survival (PFS) of different pathological staging. Univariate and multifactorial Cox risk proportional regression analyses were performed to compare the relationship between QPRT expression and each clinicopathological feature with breast cancer survival for BRCA. The R package “RMS” was also used to plot nomograms for 1, 3, and 5 year survival rates.

### 2.4. Immunohistochemistry (IHC) to Detect Protein Expression

Immunohistochemical staining results of QPRT protein in breast cancer tumor tissues and normal tissues were compared using human Protein Atlas (HPA) database.

### 2.5. Functional and Pathway Enrichment Analyses

Based on the median expression of QPRT in breast cancer for high and low expression groups, gene ontology (GO) and Kyoto Encyclopedia of Genes and Genomes (KEGG) functional and pathway enrichment analyses were performed using the R package clusterProfiler. GSEA was used to demonstrate the activation or repression of biological pathways mediated by QPRT expression [18] and was performed using the R package clusterProfiler to search for potential biological mechanisms of QPRT in breast cancer. Biological pathway enrichment of high and low QPRT expressions was analysed using the Reactome gene sets in GSEA.

### 2.6. Cell Culture and Transfection

Human breast cancer MDA-MB-231 cell line (ATCC^®^ HTB-26^™^) was cultured in Dulbecco's Modified Eagle Medium (DMEM-high glucose, 01-052-1A, Biological Industries, Beit HaEmek, Israel) containing 5% FBS (04-001-1A, Biological Industries, Beit HaEmek, Israel) and 4 mM glutamic acid base, incubated at 37°C in a humidified atmosphere of 5% CO_2_. Lentivirus vector is constructed (objective: HBLV-h-QPRT-ZsGreen-PURO, control: HBLV-h-ZsGreen-PURO), plasmid extraction kit (DP117, TIANGEN BIOTECH CO., LTD, China) was used to extract the plasmids, and 293T cells were co-transfected with Lipofectamine 3000 kit (L3000001, Thermo, USA). After infection, MDA-MB-231 cells were infected with the virus supernatant. After infection, the fusion rate of cells reached 80–90%, the cells were transferred to petri dishes, and 0.5 *μ*g/mL puromycin was added to screen positive cells under pressure. When the fluorescence rate and survival rate of cells were better than 95%, cell lines with stable expression were obtained.

### 2.7. Western Blotting (WB)

Cells were lysed in RIPA buffer (50 mM Tris-HCl, pH 7.4, 150 mM NaCl, 1% sodium deoxycholate, 1% NP-40, 0.1% SDS, 100 mM PMSF, 1 mM pepstatin A, and 1 mM E64). The released proteins were separated on an 8–12% SDS polyacrylamide gel, transferred to a PVDF membrane (IPFL00010, Millipore, Burlington, MA, USA), and treated with a primary antibody. The specific primary antibodies are as follows: QPRT (ab171944, rabbit monoclonal antibody, dilution 1 : 1000), Akt (ab8805, rabbit polyclonal antibody, dilution 1 : 500), P-Akt (ab38449, rabbit polyclonal antibody, dilution 1 : 500), PI3K (ab32089, rabbit monoclonal antibody, dilution 1 : 1000), P-PI3K (ab278545, rabbit monoclonal antibody, 0.5 *µ*g/ml), MDM2 (ab16895, mouse monoclonal antibody, used at an assay-dependent concentration), P-MDM2 (ab170880, rabbit monoclonal antibody, dilution 1 : 50000), and *β*-actin (ab8226, mouse monoclonal antibody, 1 *µ*g/ml). Then the primary antibody was incubated at 4°C overnight, and the TBST buffer (100 mM TrIS-HCl, pH 7.5, 150 mM NaCl) was oscillated and washed for 3 times, 5 minutes each time. The second antibody was incubated at room temperature for 1 h, and the film was washed 3 times with TBST oscillation for 5 minutes each time. After the membrane was incubated with TMB substrate for 1 minute, the membrane was soaked in developing solution until the strip was clear and cleaned with tap water. Then the membrane was fixed with fixing solution, and the imaging was observed with a gel imaging analysis system (XR+, Bio-RAD Laboratories, China).

### 2.8. Statistical Analysis

Statistical analysis was carried out using SPSS software (version 20.0, SPSS Inc., Chicago, IL, USA). Data were expressed as mean ± SD. Student's *t*-test was employed to determine *p* values. The *χ*^2^ test and Fisher's exact test were employed to assess the association between factors. Survival curves were created by the Kaplan–Meier method and compared by the log-rank tests. Multivariate survival analysis was conducted with the multivariate Cox proportional hazard regression model. Significant difference was recognized at *P* < 0.05.

## 3. Results

### 3.1. QPRT Was Significantly Expressed in Most Tumour Tissues

**Figure 1:**
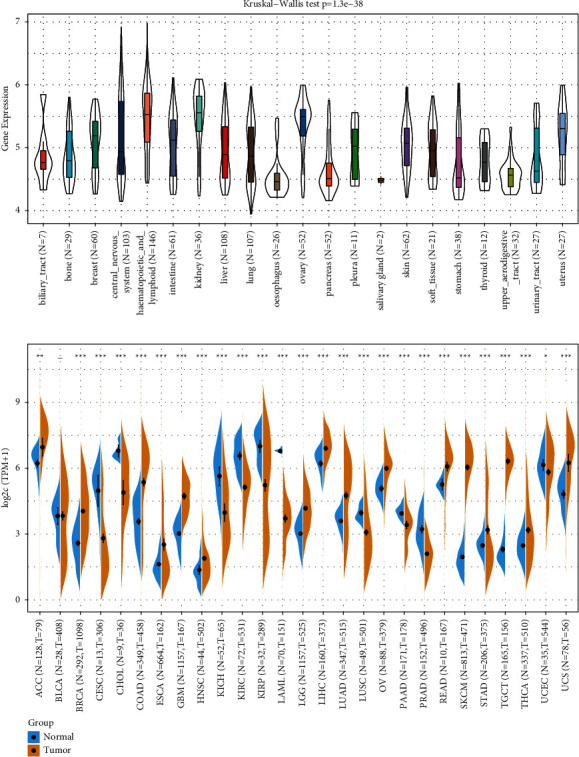
Expression levels of QPRT in different tumours: (a) QPRT expression in the CCLE dataset, total 21 tumour cell lines; (b) QPRT expression in the integrated GTEx and TCGA dataset, total 27 tumors. ^*∗*^*P* < 0.01, ^*∗∗*^*P* < 0.001, and ^*∗∗∗*^*P* < 0.0001.

Analysis of the CCLE dataset showed that QPRT was significantly expressed in all 21 tumour cell lines (Figure 1(a)). Integration of data in TCGA and GTEx revealed upregulation of QPRT expression in 16 tumours, including BRCA, COAD, and GBM, and downregulation in 10 tumours, including CHOL, KICH, and KIRP, among 27 tumour types.

### 3.2. QPRT Was Associated with the Prognosis of Certain Tumours

**Figure 2:**
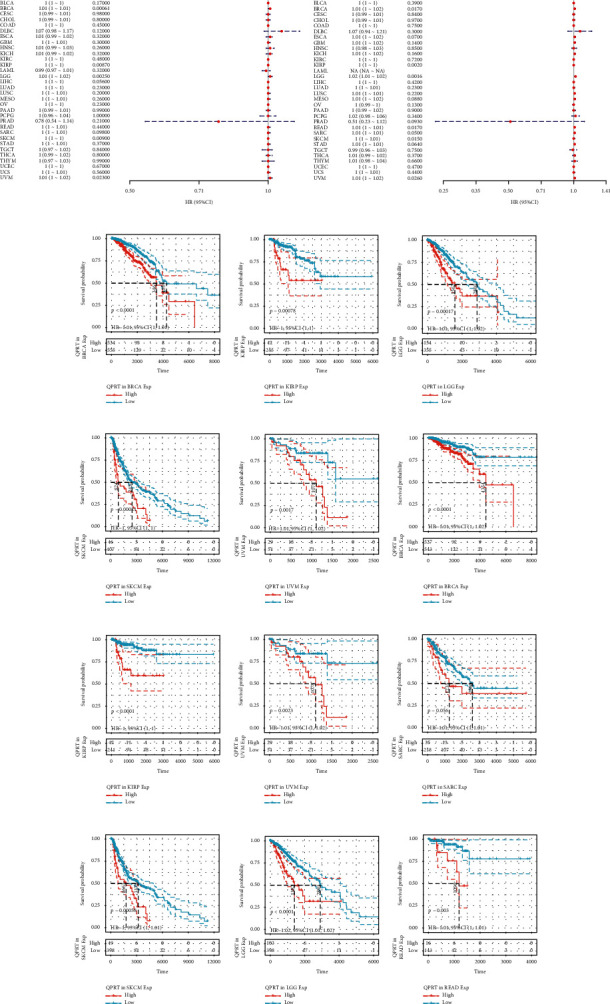
OS and DSS of high and low expressions of QPRT in each tumour: (a) forest plot of OS of QPRT in each tumour; (b) forest plot of DSS of QPRT in each tumour; (c–g) KM survival curve to show the OS of patients with high and low QPRT expressions in different tumors; (h–n) KM survival curve to show the DSS of patients with high and low QPRT expressions in different tumors.

High QPRT expression was significantly associated with poor OS prognosis in patients with BRCA, KIRP, LGG, SKCM, and UVM, and the relationship between high and low QPRT expressions and patients with each tumour was further confirmed using KM curves (Figures 2(a) and 2(b)). To avoid the impact of nontumour death during follow-up, the relationship between QPRT expression levels and prognostic DSS (disease-specific survival) was analysed, and QPRT was found to be prognostically significant only with BRCA, KIRP, LGG, and READ tumours (Figure 2(c)). QPRT was hypothesized to be a prognostic marker for tumour DSS based on the KM curve (Figure 2(d)).

### 3.3. QPRT Expression Was Significantly Associated with Different Pathological Staging of BRCA

**Figure 3:**
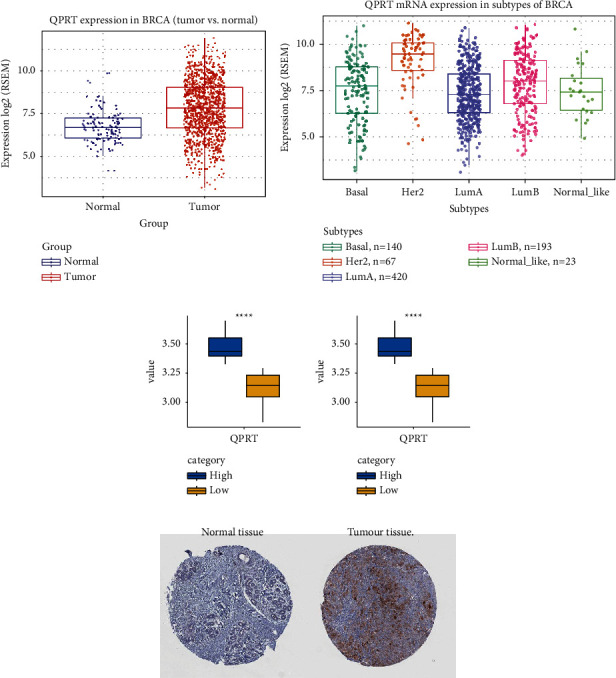
The expression of QPRT in BRCA: (a) differential expression of QPRT in BRCA tumour tissues and normal tissues; (b) expression of QPRT in different pathological staging of BRCA; ((c), (d)) the high and low expression distribution of QPRT in HER2 breast cancer, the data come from GSE46563 and GSE59246, respectively; (e) breast cancer immunohistochemical map.

QPRT expression was significantly correlated with poor prognosis in BRCA patients, and QPRT expression was significantly higher in BRCA tumour tissues than in normal tissues (Figure 3(a)). Subsequent analysis of expression in different pathological subtypes of BRCA revealed significant differences in QPRT expression in all pathological subtypes, with the highest expression in the HER2 type (Figure 3(b)). The data analysis in the two validation sets (GSE46563 and GSE59246) revealed a significant differential expression of QPRT in HER2 breast cancer (Figures 3(c) and 3(d)). The immunohistochemical results (Figure 3(e)) showed that QPRT was localized in the cytoplasm, cell membrane, and nucleus and showed a positive expression in pathological breast cancer tissues but not in normal tissues.

### 3.4. The Prognosis of BRCA Was Significantly Associated with Many Factors

**Figure 4:**
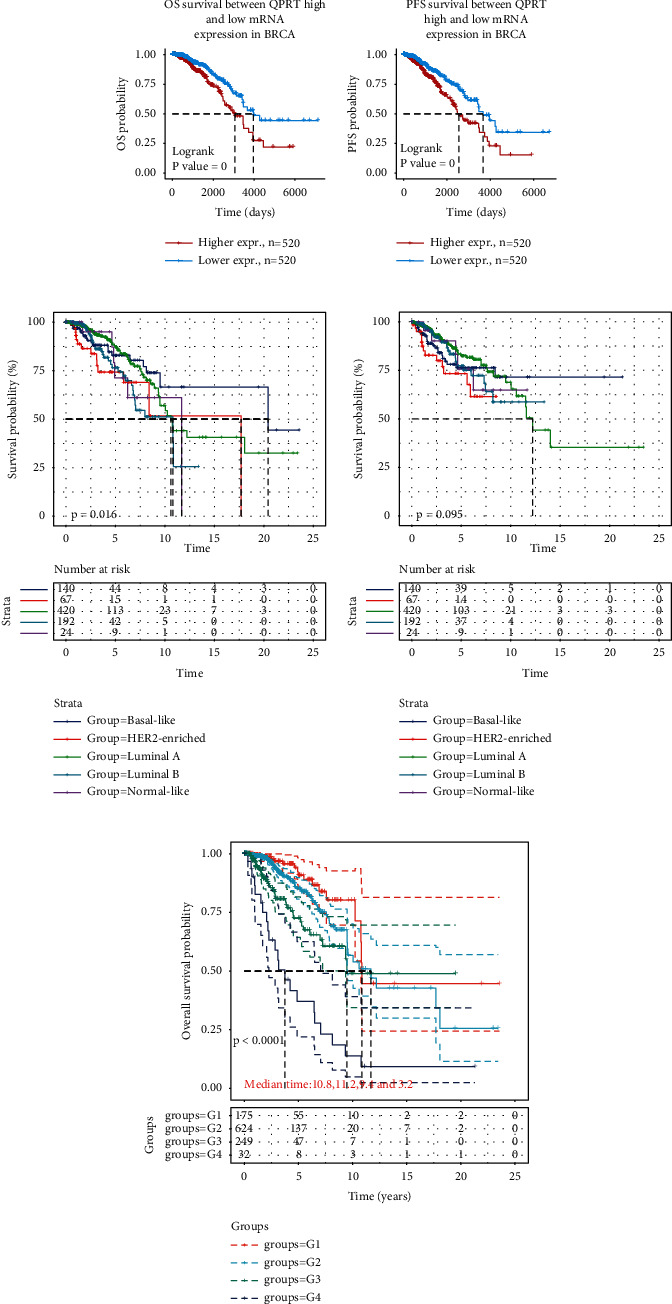
Prognostic relationship between QPRT and BRCA: (a) the relationship between high and low QPRT expressions and OS of BRCA by KM survival analysis; (b) the relationship between high and low QPRT expressions and PFS of BRCA by KM survival analysis; (c) the relationship between QPRT and OS of different pathological staging of BRCA; (d) the relationship between QPRT and PFS of different pathological staging of BRCA; (e) the relationship between QPRT and OS of different pTNM staging of BRCA.

Survival analysis showed that breast cancer patients with low QPRT expression had higher OS and PFS than those with high expression (Figures 4(a) and 4(b)). Survival analysis of different pathological staging of BRCA also showed that the median survival time was significantly longer in basal-like and HER2-enriched than in luminal A, luminal B, and normal-like (Figure 4(c)). In addition, high QPRT expression was significantly associated with the prognosis of breast cancer patients with different pTNM stages (Figure 4(e)).

**Figure 5:**
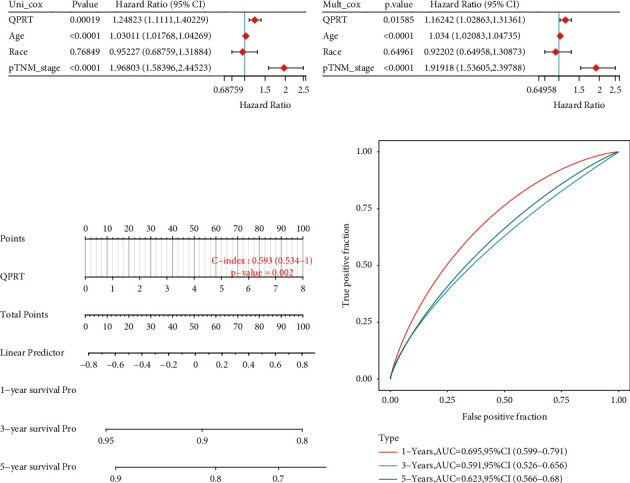
Prognosis of the BRCA prediction column line graph: (a) single-factor Cox regression analysis of the risk relationship of BRCA with age, race, and pTNM; (b) multifactor Cox regression analysis of the risk relationship of BRCA with age, race, and pTNM; (c) column line graphs for predicting the overall survival of BRCA patients at 1, 3, and 5 years; (d) ROC curve graphs for overall survival at 1, 3, and 5 years.

We also developed a prognostic model for BRCA to assess the impact of each factor on survival. In univariate survival analyses, BRCA cases with high QPRT expression had a poor OS. In Cox risk proportional regression analysis, after adapting for age, grade, tumour size, and subtype, QPRT was still an independent prognostic factor for OS (Figures 5(a) and 5(b)). Columnar tables were established to predict the prognostic survival of breast cancer patients at 1, 3, and 5 years, and the ROC curves showed that the 1-year (AUC = 0.695, 95% CI: 0.599–0.791) survival prediction model was the best model (Figures 5(c) and 5(d)).

### 3.5. QPRT May Be Involved in BRCA Progression through the PI3K/Akt Signalling Pathway

**Figure 6:**
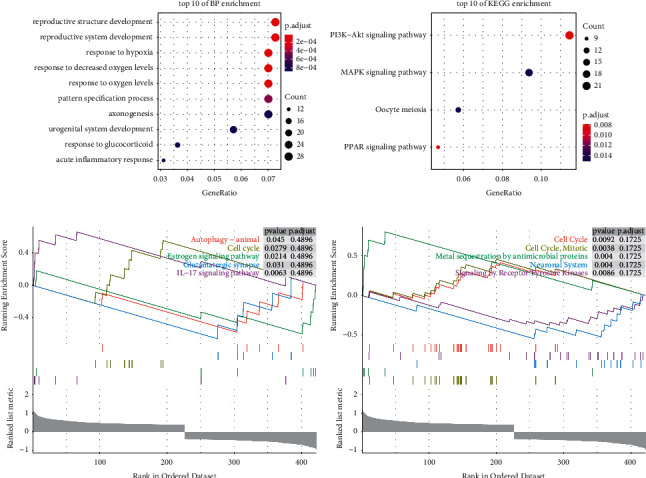
BRCA whole gene enrichment analysis: (a) enrichment of GO function in QPRT expression; (b) enrichment of KEGG function in QPRT expression; (c) enrichment of the KEGG pathway in GSEA with high and low QPRT expressions; (d) enrichment of biological processes in the Reactome gene set with high and low QPRT expressions. *P* < 0.05 is statistically significant.

Enrichment analysis revealed (Figures 6(a) and 6(b)) that oxygenation levels and the development of the reproductive system were the most significant biological functions; the PI3K-Akt signalling pathway was the most significant KEGG pathway; some of the related functions and pathways are listed in Table 1. Subsequent GSEA showed that the oestrogen signalling pathway and cell cycle were the most significant KEGG pathways and that the cell cycle and mitosis were the most significantly related biological processes in the Reactome gene set (Figures 6(c) and 6(d)).

### 3.6. QPRT Activated the PI3K/Akt Signalling Pathway in Breast Cancer Cells

**Figure 7:**
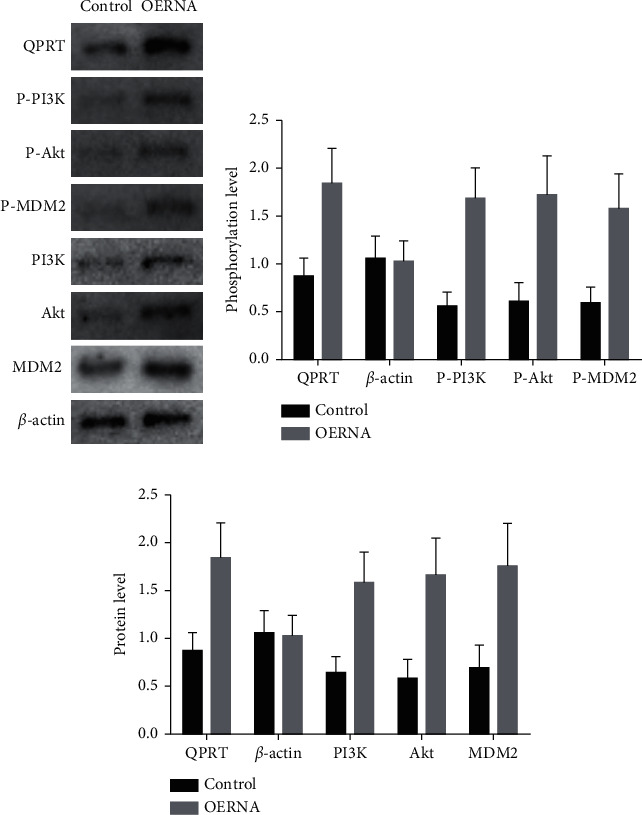
QPRT overexpression induces phosphorylation of PI3K/Akt-related signalling pathways: (a) western blot; (b) phosphorylation levels of different signalling pathways; (c) protein levels of different signalling pathways.

We confirmed the influence of QPRT on the PI3K/Akt signalling pathway by western blotting (Figure 7). Phosphorylation and protein levels of P-PI3K (ab278545) and P-Akt (ab38449) were significantly increased in MDA-MB-231 cells with foreign expression of QPRT. We also evaluated the PI3K/Akt downstream protein kinase P-MDM2 (ab170880) and found a significant enhancement of its phosphorylation and protein levels. These results indicate that QPRT may promote breast cancer progression through the PI3K/Akt pathway.

## 4. Discussion

Cancer is a significant factor affecting the health and longevity of people worldwide, with nearly 20 million new cancer cases and nearly 10 million deaths reported worldwide in 2020 [19]. Pan-cancer analysis can identify the similarities and differences in the tumour genomes and provide helpful information for cancer diagnosis and treatment [20]. In the present study, we evaluated the efficacy of QPRT for pan-cancer analysis. QPRT is significantly expressed in most tumours, and its expression is related to prognosis. Among them, the expression of QPRT has a prominent effect on the prognosis of breast cancer patients.

Because of the strong relationship found for QPRT in breast cancer, this study mainly analysed the relationship between QPRT and breast cancer. The TCGA dataset as the test set revealed significant QPRT expression in breast cancer, especially HER2 breast cancer. These results were reproduced in two GEO validation sets. A study screening for prognosis-related candidate genes in breast cancer showed that QPRT expression was significantly associated with prognosis in breast cancer patients [21]. The analysis in the present study shows that QPRT is an independent prognostic factor of breast cancer and is related to different pathological subtypes.

In breast cancer, HER2 gene amplification can lead to the proliferation of specific aggressive breast cells, and HER2 expression has been identified as an independent factor for the poor prognosis of breast cancer patients [22]. Targeted therapy is one of the treatments aimed at improving the survival rate of HER2-positive breast cancer patients, but the selection of targeted genes still needs further study [23].

QPRT catalyses the production of nicotinic acid mononucleotide (NMN), which in turn promotes the synthesis of nicotinamide adenine dinucleotide (NAD+), which plays a crucial role in cell survival [24]. Zhang et al. used in vivo and in vitro experiments to confirm that QPRT promotes growth, migration, and invasion of breast cancer and inhibits cell apoptosis [17]. Liu et al. also provided strong evidence that upregulation of QPRT promotes breast cancer progression [16]. Earlier work indicated that QPRT might have an antiapoptotic function (Ullmark et al., 2017). Furthermore, QPRT was identified as a potential prognosis biomarker of BC [21]. However, whether QPRT is an independent prognostic factor in invasive breast cancer and the mechanisms by which QPRT may contribute to invasive breast cancer remain undefined. Thus, the present study was based on this previous research and aimed to explore the mechanism underlying promoting breast cancer progression by QPRT.

QPRT overexpression is known to activate the PI3K/Akt signalling pathway in cancer cells [25], but this has not been proven in breast cancer. The GSEA results presented here showed that QPRT expression was related to the PI3K/Akt signalling pathway, and western blot analysis showed that overexpression of QPRT can increase the phosphorylation levels of PI3K and Akt, indicating that QPRT and the PI3K/Akt signalling pathway may have a positive feedback effect in breast cancer.

Phosphoinositide 3-kinase (PI3K) can integrate signals from growth factors, cytokines, and other extracellular stimuli, and the modification of this pathway is closely related to the pathogenesis of cancer [26, 27]. Protein kinase B (PKB, also known as Akt) is an essential mediator of the PI3K pathway and the signalling endpoint of various growth factors and cytokines [28]. The PI3K/Akt signalling pathway is one of the phosphatidylinositol signalling systems involved in tumorigenesis, cell growth, proliferation, metabolism, survival, and apoptosis [29]. The PI3K/Akt signalling pathway is activated in various cancers and has been proven to be one of the most important signalling pathways in cancer development [30]. The PI3K/Akt signalling pathway has attracted increasing attention in breast cancer research as activating this pathway can promote breast cancer cell proliferation, inhibit apoptosis [31], and modulate cell invasion [16]. Human epidermal growth factor receptor-2 (HER2) is involved in the development of breast cancer through the PI3K/Akt/mTOR pathway [27], and the PI3K/Akt/mTOR pathway is an important pathway involved in chemoresistance and survival of triple-negative breast cancer (TNBC) [32]. However, the current study also had some limitations. This research was based on microarray data analysis. The samples from the datasets were insufficient and without cancer stage information, and data from biological samples carried out no confirmation. Consequently, large-scale, potential, and widespread clinical examinations are required to confirm our results. It was necessary to obtain a single gene to profile QPRT expression in BRCA. The mechanism of QPRT needs further findings through in vivo and in vitro models.

## 5. Conclusions

Collectively, our results here support a vital role for QPRT in breast cancer and indicate that its upregulation is related to the poor prognosis of patients with BRCA. Subsequently, in vitro experimental results show that QPRT upregulation may affect breast cancer progression by activating the PI3K/Akt signalling pathway. The current study implies that QPRT may therefore be a novel specific therapeutic target for breast cancer treatment.

## Figures and Tables

**Table 1:** The most significant GO biofunction and KEGG pathways in the enrichment analysis.

Class	Function/pathway	*p* value	*p*. adjust	*q*-value
BP	Response to hypoxia	7.92E-09	3.08E-05	2.57E-05
	Response to decreased oxygen levels	1.50E-08	3.08E-05	2.57E-05
	Response to oxygen levels	5.54E-08	7.58E-05	6.32E-05
	Reproductive structure development	9.59E-08	9.10E-05	7.58E-05
	Reproductive system development	1.11E-07	9.10E-05	7.58E-05
	Pattern specification process	6.59E-07	0.000450942	0.00037565
	Urogenital system development	1.52E-06	0.000852121	0.000709846
	Axonogenesis	1.66E-06	0.000852121	0.000709846
	Response to glucocorticoid	2.04E-06	0.000874411	0.000728415
	Acute inflammatory response	2.34E-06	0.000874411	0.000728415
KEGG	PI3K-Akt signalling pathway	3.00E-05	0.007825176	0.007132445
	PPAR signalling pathway	7.00E-05	0.009139327	0.00833026
	MAPK signalling pathway	0.000198423	0.014943102	0.01362025
	Oocyte meiosis	0.000229013	0.014943102	0.01362025

## Data Availability

The data used to support the findings of this study are available from the corresponding author upon request.
